# A Global Digital Citizen Science Policy to Tackle Pandemics Like COVID-19

**DOI:** 10.2196/19357

**Published:** 2020-05-26

**Authors:** Tarun R Katapally

**Affiliations:** 1 University of Regina Regina, SK Canada

**Keywords:** COVID-19, pandemic, citizen science, smartphones, population health, mHealth, eHealth, big data, virus, infectious diseases, public health, digital epidemiology

## Abstract

The coronavirus disease (COVID-19) pandemic is an extremely complex existential threat that requires cohesive societal effort to address health system inefficiencies. When our society has faced existential crises in the past, we have banded together by using the technology at hand to overcome them. The COVID-19 pandemic is one such threat that requires not only a cohesive effort, but also enormous trust to follow public health guidelines, maintain social distance, and share necessities. However, are democratic societies with civil liberties capable of doing this? Mobile technology has immense potential for addressing pandemics like COVID-19, as it gives us access to big data in terms of volume, velocity, veracity, and variety. These data are particularly relevant to understand and mitigate the spread of pandemics such as COVID-19. In order for such intensive and potentially intrusive data collection measures to succeed, we need a cohesive societal effort with full buy-in from citizens and their representatives. This article outlines an evidence-based global digital citizen science policy that provides the theoretical and methodological foundation for ethically sourcing big data from citizens to tackle pandemics such as COVID-19.

## A Pandemic in a Digital World

When our society has faced existential crises in the past, we have banded together to overcome the challenge. The coronavirus disease (COVID-19) pandemic is one such threat that requires not only a cohesive effort, but also enormous trust to follow public health guidelines, maintain social distance, and share basic necessities [1,2]. Are democratic societies with civil liberties capable of doing this?

The answer to this question is perhaps right in our pockets. The internet connects us all, and with more than 3 billion devices currently in circulation worldwide [3], if there is one ubiquitous tool that leverages the power of the internet like no other, it is the smartphone. The idea of smartphones being one of the primary solutions to this global problem might seem far-fetched until we unpack its potential. Smartphones provide us with the ability to directly reach and engage with a significant proportion of the world’s population in near real time, which has immense potential for addressing COVID-19 outbreaks via rapid detection. Moreover, smartphones have the capacity to provide big data via sensors, such as global positioning systems [4].

Smartphones can provide data about population movement patterns [5], which are extremely pertinent to not only understand and implement social distancing and isolation measures, but also to develop predictive epidemiological modeling of virus spread. In addition, smartphones can facilitate real time interventions to modify behavior and link people with health care services—aspects that can be used to manage both the physical and mental health effects of COVID-19. However, for such intensive and intrusive data collection measures to succeed, we need buy-in from citizens.

The policies and responses of governments around the world have varied in speed and intensity [6], but what unites them is the evidence that COVID-19 is highly contagious. The reality is that the success of government policies to detect, contain, and minimize the spread of COVID-19 lies beyond the health care systems that are currently barely coping with the ever-increasing growth of COVID-19 positive cases [7]. The success of government policies ultimately depends on the willingness of citizens to follow public health guidelines and abide by laws restricting free movement, which is a challenge in free societies where citizens in one country might abide by regulations better than citizens in another country [8,9]. Thus, whether it is the ability to leverage ubiquitous digital tools such as smartphones, or whether it is the success of government policies to manage and minimize the COVID-19 outbreaks, implementation of these strategies ultimately depends on citizen engagement. The key to building public trust is to link citizen science to citizen engagement.

## The Digital Citizen Science Policy Solution

Citizen science is a participatory approach that can range from contributory and collaborative methods (data collection and analysis) to cocreation of knowledge (conceptualization and knowledge translation). It can pave the way for increased citizen engagement and crowdsourcing of big data during this crisis. Citizen science has become increasingly interdisciplinary over the past couple of decades [10], which has implications for population health science—a field of science that plays a key role in addressing broad health inequities [11]. Moreover, with the increasing power of citizens to affect change, citizen science is earning a place in national science policies of countries such as the United States and Australia by complementing the efforts of governments and health professionals [10].

Citizen science offers an opportunity to transform population health science by engaging a larger proportion of the population in data collection to bring citizen perspectives closer to traditional decision-making processes [10]. However, there is currently no coherent citizen science policy to tackle the COVID-19 pandemic. The success of citizen science depends on innovative mobile health (mHealth) apps. The ultimate purpose of mHealth and citizen science, particularly from a disease risk management point of view, is to enable digital epidemiological modeling to prevent, detect, and manage the current wave of the COVID-19 pandemic, as well as to predict and prepare for subsequent waves. One of the biggest challenges in the current COVID-19 pandemic has been the inability to effectively track the virus and population movements that facilitate the spread of the disease.

Digital epidemiology is a novel field of science that has been growing rapidly in the past few years. It has been fueled by the increasing availability of data and computing power, as well as by breakthroughs in data analytics [12,13]. The digital health revolution has transformed the collection and analysis of electronic health records, as well as physiological and behavioral measurements at the individual level. However, our health care systems are primed for benefiting downstream service providers for disease management rather than promoting upstream policies to prevent disease development. Another concern is the lack of ethical engagement in digital health, where the power resides predominantly with researchers and providers.

These challenges point toward the need for policy that addresses the intersection of citizen science, innovation, and health to facilitate mHealth and digital citizen science platforms that enable ethical surveillance, integrated knowledge translation, and real time interventions [14,15]. This combination is critical to address population health crises such as the COVID-19 pandemic. The creation of mHealth and digital citizen science platforms can enable:

- Implementation of ethical real time surveillance to assess COVID-19 community risk by subjective and objective (sensor-based) longitudinal data gathering.
- Implementation of near real time integrated knowledge based on the data provided by crowdsourced citizens to increase awareness of health-promoting practices.
- Implementation of evidence-based real time communication that not only link citizens to health care services but also ensures safe social distancing and psychological support.
- Development of decision-making dashboards at the back end, which enhances real time information sharing and data analytics that would inform community decision making to mitigate risk and ensure productivity during health crises.
- Addressing future pandemics by developing models of risk mitigation, which would not only prepare communities, but also enhance health system capacity for future communicable diseases.

**Figure 1:**
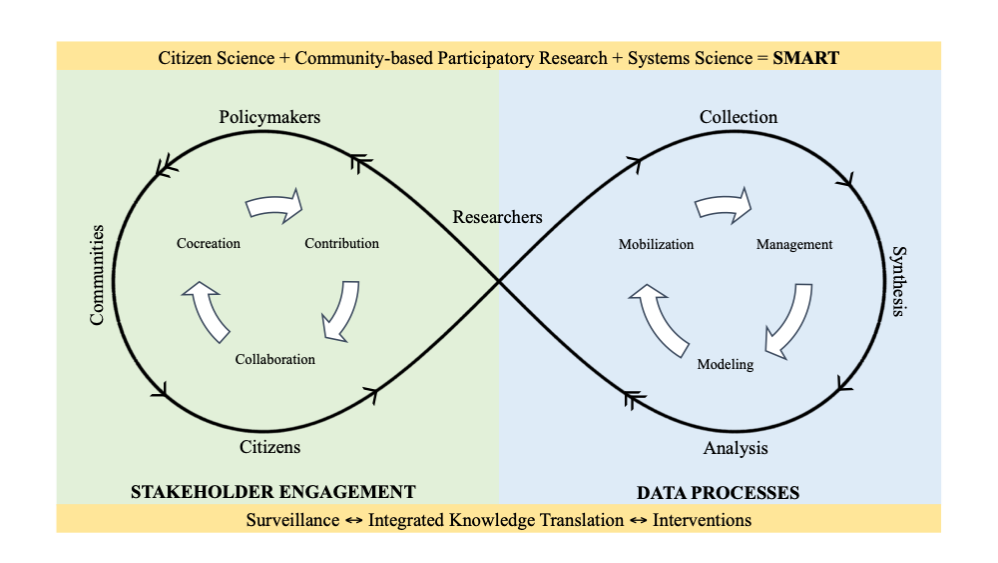
The SMART Framework.

The development of such platforms requires robust theoretical underpinnings that provide stakeholders a clear pathway to decision making. One such pathway is the SMART Framework [11]. It integrates citizen science, community-based participatory research, and systems science through ubiquitous tools to conduct population health research in the digital age (Figure 1). A key component of this framework is the necessity to repurpose citizen-owned ubiquitous communication devices (ie, smartphones) that have revolutionized the ability to sense, share, and link big data. This potential for repurposing smartphones is magnified in the current COVID-19 crises because smartphones have the reach to create equity by empowering disenfranchised citizens, and smartphone-based apps have the capacity to source big data to inform policies through the voice of the citizens.

## Operationalizing Digital Citizen Science Policy

The flexibility of mHealth and digital citizen science platforms to scale-up opens enormous opportunities to operationalize policy across jurisdictions, whether within a single country or across a cohort of nations. For instance, from a Canadian perspective, health care implementation is the mandate of provinces and territories [16], so natural authorities to introduce digital citizen science policies would be the provinces and territories, where resources are obtained from health care budgets. The federal governments can play a larger role in mandating such policies, which will eventually play a part in enrolling entire populations, a critical factor in the success of mHealth and digital citizen science platforms.

However, digital citizen science policies can exist outside traditional health care systems, as this approach takes a population health lens of health in all policies, which has its own advantages of systems integration [17]. Because citizen science platforms can exist outside health care systems, they do not have to exist within government ecosystems.

In fact, mHealth and digital citizen science platforms have traditionally been used for noncommunicable disease monitoring and interventions, which are implemented outside health care systems by academic research units [14,15]. As the same approach can be used to tackle pandemics like COVID-19, there are opportunities to commercialize mHealth and digital citizen science platforms that will break the silos of jurisdictional authorities. One scenario is that big technology companies, which already have access to a significant proportion of big data collected passively from smartphones across the world, will become major players in disease management [18]. The pros and cons of such a scenario need to be debated comprehensively and are beyond the scope of this policy document. However, the opportunity to commercialize and introduce mHealth and digital citizen science platforms allows groups at risk, such as Indigenous communities, to take control and tackle the risk of pandemics such as COVID-19 [19]. Thus, although the operationalization digital citizen science policies can take many shapes and forms, it is apparent that government agencies will lose their grip on digital health if they do not act now [20].

## Benefits of Citizen-Focused Digital Health

Apart from the obvious benefits to the health of populations by reducing the risk of communicable diseases like COVID-19, when digital health takes a citizen science approach, it can enable community empowerment by connecting citizens for a common cause, manage misinformation by directly engaging citizens, and inform evidence-based decision making using big data. There are multiple benefits that would result from linking citizen science and citizen engagement.

The social and societal benefits are:

- Community empowerment: citizen science connects citizens with one another, facilitating engagement, knowledge sharing, and problem solving, especially during existential crises.
- Decision making: citizen science can bring citizens closer to traditional decision-making processes by contributing to the greater dialogue around issues that are of the highest concern to society.
- Misinformation management: citizen science can play an important role in countering digital misinformation.

The population health benefits are:

- Prevention, early detection, and policy interventions: this approach would engage citizens in real time to predict and address global population health risks such as the COVID-19 outbreak, as well as link these risks with upstream policy and downstream health care use data to develop preventive public health policies.
- Evaluating population health interventions: a digital citizen science approach provides the ability to evaluate population health interventions in different jurisdictions, which are especially pertinent when different and incohesive municipal, provincial, and federal policies to counter a pandemic such as COVID-19 are being implemented worldwide.
- Real time interventions: the ability to engage with citizens in real time to deploy behavioral interventions that can help manage population health crises such as COVID-19 are critical to implement measures such as social distancing and mental health support for self-isolating populations across the world.

The benefits to decision makers are:

- Development of decision-making dashboards: real time big data can be used to develop decision-making dashboards that incorporate advanced data analytics to inform global policies during pandemics and manage productivity during social distancing, if necessary.
- Assessing environmental effects: using citizen science, we can understand the effect of physical, social, and cultural environments on health behaviors such as social distancing and abiding by public health guidelines, aspects that are essential to flatten the curve of pandemics.
- Identifying and addressing environmental risks: the trajectory of pandemics such as COVID-19 need to be addressed by taking into consideration varied environmental risks in different geographic regions of the world, and citizen scientists could play an important role in enabling global risk management of pandemics.

The economic benefits are:

- Licensing user access: mHealth and digital citizen science platforms can be licensed to external users on a large-scale to enable commercially viable digital epidemiological studies worldwide.
- Developing mHealth smartphone apps: similarly, mHealth and citizen science platforms can be the source of commercially viable front end mHealth smartphone apps that can be developed rapidly to tackle population health crises such as pandemics.
- Commercialization of dashboards: there is the potential to commercialize decision-making dashboards for different jurisdictions from mHealth and digital citizen science platforms to facilitate real time information sharing worldwide.

## Anticipating and Addressing Challenges

### Data Privacy and Security

One of the biggest challenges to deploying citizen science at any scale is individuals’ right to privacy. As smartphone-based citizen scientist data are granular and detailed owing to the leverage of sensors such as global positioning systems, protecting privacy and anonymity of citizens through strong encryption processes must be the highest priority [11,21,22]. Moreover, before any citizen scientist engagement, obtaining informed consent has to be mandatory. Apart from obtaining informed consent, citizen scientists should be provided an option to dropout and delete their own data (ie, embedding of open source features as much as possible). However, to truly conduct ethical surveillance, data co-ownership is essential, where citizen scientists are able to participate in data visualization, analysis, and knowledge translation [9,12,13]. Ethical surveillance is paramount as COVID-19 tracking escalates across the world because the elimination of ethical data collection now can potentially open doors for invasive surveillance in the future [23]. Apart from equitable engagement with citizens, ethical surveillance also depends on advanced encryption and secure server storage processes, which abide by jurisdictional laws. These capabilities need to be embedded into digital platforms for citizen science to ensure the data that can lead to citizen identification is secured with rigorous ethical and analytical protocols. More importantly, it should be made clear to citizens that anonymity is the guiding principle behind all data privacy and security procedures.

### Data Validity and Linkages

Data validity eventually depends on successful and consistent citizen science engagement with digital tools that facilitate secure data collection, synthesis, analyses, and dissemination [24]. A significant factor in citizen scientist engagement would be intuitive front-end human-computer interfaces such as easy to use smartphone apps, and back-end databases that encapsulate data management, modeling, and mobilization. An important feature in ensuring the success of digital citizen science platforms is citizen contribution to the development of both front-end apps and back-end decision-making dashboards. Open-source back-end data management systems [25] can facilitate collaboration across sectors and disciplines via linkages with other open-source databases to maximize the potential of citizen science [26-29]. Nevertheless, the future of citizen science platforms in digital health is dependent on linking upstream administrative, policy, and behavioral citizen data that potentially exist outside the health care system with downstream health care use data. Ultimately, these data linkages require not only legislative action but also digital and logistical infrastructure.

### Internet Inequity

The most systemic barrier to digital citizen science is internet inequity. Internet inequity is defined as differential internet access based on wealth, location (urban, rural, or remote), gender, age, or ethnicity [11]. Thus, a risk of the digital citizen science approach is the potential to widen existing health disparities by excluding vulnerable populations who do not have access to the internet [30]. With respect to smartphone dependence, evidence indicates that minority groups and younger, lower income, and less-educated users are more likely to be dependent on smartphones to access the internet [31], an inequity that could be turned into an opportunity via mHealth and digital citizen science approaches. The digital divide is a complex phenomenon, with varying internet bandwidth across different low-, middle-, and high-income countries. However, there seems to be a connection between bandwidth divide and income divide worldwide [31]. Ultimately, as the United Nations has declared that access to the internet is a human right [32], policy makers have a moral responsibility to address internet inequity, and crises such as the COVID-19 pandemic should provide the impetus for internet equity.

### Legitimization and Citizen Scientist Compliance

As the COVID-19 experience has demonstrated, pandemics present public health, economic, and social challenges on a global scale unlike anything else we have witnessed. Thus, tackling pandemics will require transformational change with significant support in terms of personnel training, funding, and time, and increases in transparency to balance the power between decision makers, researchers, and citizens. The COVID-19 challenge provides the prospect of overturning the traditional neglect that citizen science has endured to offer new insights for solving population health crises [33]. However, the ultimate success of citizen science is dependent on effective engagement. The challenges of citizen scientist recruitment, retention, and compliance need to be addressed by a combination of logistical, technological, and methodological solutions before, during, and after data collection.

The integration of citizen science with community-based participatory research can aid citizen engagement and empowerment [12]. Another important factor is developing strategies that are specific to different cohorts, demographic groups, and jurisdictions by taking into consideration historical, cultural, and sociopolitical contexts of populations. Nevertheless, the big question is, regardless of the need for a cohesive societal effort to overcome pandemics, why will citizens ultimately comply? Transformational change requires radical ideas, and if we want citizens to trust institutions, perhaps there is a need to provide citizens with incentives for ethical surveillance. In other words, pay people for sharing data that will enable us to understand the progression of pandemics, and enforce the stringent public health measures necessary to flatten their curve. We cannot secretly monitor citizens; at the same time, we are in uncharted waters when it comes to health and economic consequences of this pandemic. Thus, it is important to seriously consider incentivizing citizens to share data, whether it is through monetization of citizen scientist data, providing tax credits, or other in-kind contributions that would not only ensure equitable participation of citizens but also aid economic equity.

## Conclusion

The COVID-19 pandemic is an extremely complex existential threat that requires cohesive societal effort to address health system inefficiencies and to overcome gaps in real time data analytics. When our society has faced existential crises in the past, we have banded together by using the technology at hand to overcome them. Pandemics such as COVID-19, although extremely challenging, offer us an opportunity to innovate and consider options that would normally be ignored due to lack of vision, resources, and coordination. The answer to the current crisis could be right in our pockets. With more than 3 billion smartphones currently in circulation worldwide, they provide us with the ability to directly reach and engage with a significant proportion of the world’s population in near real time. However, for such intensive and potentially intrusive data collection measures to succeed, we need a cohesive societal effort with significant buy-in from citizens. A global policy for digital citizen science that facilitates citizen engagement across the world via sophisticated digital epidemiological platforms is a viable solution to not only overcome the COVID-19 outbreak but also better anticipate, prepare, and tackle future pandemics with the rapid response that is necessary in our globalized world.

## Abbreviations

COVID-19: coronavirus disease
mHealth: mobile health
